# Kidney Damage in Long COVID: Studies in Experimental Mice

**DOI:** 10.3390/biology12081070

**Published:** 2023-07-30

**Authors:** Rajalakshmi Ramamoorthy, Hussain Hussain, Natalia Ravelo, Kannappan Sriramajayam, Dibe M. Di Gregorio, Kodisundaram Paulrasu, Pingping Chen, Karen Young, Andrew D. Masciarella, Arumugam R. Jayakumar, Michael J. Paidas

**Affiliations:** 1 Department of Obstetrics, Gynecology and Reproductive Sciences, University of Miami Miller School of Medicine, Miami, FL 33136, USA; rxr1310@med.miami.edu (R.R.); natalia.ravelo@jhsmiami.org (N.R.); 2 Department of Internal Medicine and Infectious Disease, Larkin Community Hospital, Miami, FL 33143, USA; hussainhussainmd77@gmail.com; 3 Department of Molecular and Cellular Pharmacology, University of Miami Miller School of Medicine, Miami, FL 33136, USA; kxs1087@med.miami.edu; 4 University of Miami College of Arts and Sciences, Coral Gables, FL 33146, USA; dmd1110@med.miami.edu; 5 Department of Surgery, University of Miami Miller School of Medicine, Miami, FL 33136, USA; kxp641@miami.edu; 6 Department of Pediatrics, University of Miami Miller School of Medicine, Miami, FL 33136, USA; pchen2@med.miami.edu (P.C.); kyoung3@med.miami.edu (K.Y.); 7 University of Miami Miller School of Medicine, Miami, FL 33136, USA; a.masciarella@med.miami.edu; 8 Department of Biochemistry and Molecular Biology, University of Miami Miller School of Medicine, Miami, FL 33136, USA

**Keywords:** fibrosis, inflammation, kidney, long COVID, SARS-CoV-2

## Abstract

**Simple Summary:**

In this study, we investigated the long-term effects of COVID-19 on the kidneys using mice infected with a similar coronavirus. We examined markers in the kidneys related to inflammation, scarring, and damage. We identified that certain markers linked to scarring and inflammation remained high in the mice’s kidneys even after the initial infection, suggesting kidney damage. However, other markers did not show significant changes (e.g., mRNA levels of TNFR-1, WFDC2, B2M, likely due to factors influencing their half-life and translation rate). To explore treatments, we tested a drug called SPIKENET (SPK), a 15-amino acid synthetic peptide, that blocks the virus from attaching and entering cells. When we gave SPK to the mice, it decreased the certain marker levels in their kidneys in both the group of mice that were treated shortly after infection and the group that received treatment a year after the infection. These findings indicate that kidney scarring may begin early in COVID-19 and that targeting specific markers and proteins with treatments like SPK could help prevent kidney damage in both early and long-term COVID-19 cases.

**Abstract:**

Signs and symptoms involving multiple organ systems which persist for weeks or months to years after the initial SARS-CoV-2 infection (also known as PASC or long COVID) are common complications of individuals with COVID-19. We recently reported pathophysiological changes in various organs post-acute infection of mice with mouse hepatitis virus-1 (MHV-1, a coronavirus) (7 days) and after long-term post-infection (12 months). One of the organs severely affected in this animal model is the kidney, which correlated well with human studies showing kidney injury post-SARS-CoV-2 infection. Our long-term post-infection pathological observation in kidneys includes the development of edema and inflammation of the renal parenchyma, severe acute tubular necrosis, and infiltration of macrophages and lymphocytes, in addition to changes observed in both acute and long-term post-infection, which include tubular epithelial cell degenerative changes, peritubular vessel congestion, proximal and distal tubular necrosis, hemorrhage in the interstitial tissue, and vacuolation of renal tubules. These findings strongly suggest the possible development of renal fibrosis, in particular in the long-term post-infection. Accordingly, we investigated whether the signaling system that is known to initiate the above-mentioned changes in kidneys in other conditions is also activated in long-term post-MHV-1 infection. We found increased TGF-β1, FGF23, NGAL, IL-18, HIF1-α, TLR2, YKL-40, and B2M mRNA levels in long-term post-MHV-1 infection, but not EGFR, TNFR1, BCL3, and WFDC2. However, only neutrophil gelatinase-associated lipocalin (NGAL) increased in acute infection (7 days). Immunoblot studies showed an elevation in protein levels of HIF1-α, TLR-2, and EGFR in long-term post-MHV-1 infection, while KIM-1 and MMP-7 protein levels are increased in acute infection. Treatment with a synthetic peptide, SPIKENET (SPK), which inhibits spike protein binding, reduced NGAL mRNA in acute infection, and decreased TGF-β1, BCL3 mRNA, EGFR, HIF1-α, and TLR-2 protein levels long-term post-MHV-1 infection. These findings suggest that fibrotic events may initiate early in SARS-CoV-2 infection, leading to pronounced kidney fibrosis in long COVID. Targeting these factors therapeutically may prevent acute or long-COVID-associated kidney complications.

## 1. Introduction

The coronavirus pandemic (COVID-19) continues to impact millions of people around the world with various ongoing morbidities and mortalities [1,2,3,4,5]. As of March 2023, there have been over 6.8 million deaths since the pandemic was declared in early 2020, with more than 39 thousand new cases reported daily worldwide. However, over 767 million individuals have successfully recovered from mild, moderate, or severe SARS-CoV-2 infections out of a total of 775 million affected individuals [1,2,6]. Despite the global efforts to develop a COVID-19 vaccine aimed at reducing mortality rates, there is no specific treatment for the viral infection [1,2,6,7,8,9]. Long-term complications continue to be observed among survivors, including defective sleep patterns, osteoporosis, subfertility, worsening diabetes, fatigue, and complications affecting various organ systems including musculoskeletal, cardiovascular, gastrointestinal, pulmonary, neurologic, and urologic [1,7,8,9,10]. With the multitude of SARS-CoV-2 genetic mutations and the diverse range of post-infection complications documented by scientists, the exact pathophysiology remains elusive [2,7,8,11]. Further, the progression of disease pathogenesis (from weeks to months) can be demarcated as signs involving multiple organ dysfunction or failure that occur in one or more episodes of post-acute SARS-CoV-2 infection (i.e., post-acute sequelae of SARS-CoV-2 infection) with or without obvious symptoms and behavioral changes.

MHV-1 infection of A/J mice produces a clinical SARS-like disease with a high mortality [12,13,14,15,16,17,18,19,20]. These mice develop severe lung injury at day 6–7 post-MHV-1 infection and 40–60% mortality from 7 to 12 days post-infection. At death, the lungs show severe interstitial pneumonitis with interstitial inflammatory reaction and significant lymphocyte and macrophage infiltrates. Examinations of the livers of MHV-1-infected A/J mice show evidence of severe hepatic congestion, as in humans with SARS-CoV-2 [21].

Since MHV severe acute respiratory syndrome coronavirus and SARS-CoV-2 share a common genus; the results obtained from MHV-1 could offer mechanistic insights into SARS-CoV-2 infection in humans with SARS-CoV-2 infection [12]. While MHV-1 in mice and SARS-CoV-2 in humans share various similarities, there are also differences, e.g., the presence of viral binding receptors, ACE2 versus carcinoembryonic antigen-related cell adhesion molecule 1 (biliary glycoprotein) (CEACAM1) [12], as well as the proteolytic cleavage of four important amino acids at the S1/S2 site of the SARS-CoV-2 spike protein [22]. However, the similarities offset the differences [23,24], and our observation of pathological functional changes, are highly comparable to humans with COVID-19.

Renal dysfunction has been observed in approximately 30% of patients following acute and long-term post-SARS-CoV-2 infection [2]. Further, deterioration of kidney function (elevation of urea and creatinine levels) has also been noted in patients with pre-existing chronic kidney injury [10,11,25,26,27]. Various studies in humans with post-SARS-CoV-2 infection showed varying degrees of acute tubular necrosis, glomerular lesions, focal segmental glomerulosclerosis, and renal infarction [28,29,30,31,32]. Many of these patients had comorbidities such as hypertensive nephrosclerosis, diabetes, and cardiovascular disease, which may contribute to the severity of kidney injury [28,29,30,31,32].

The exact pathophysiology of renal injury following SARS-CoV-2 infection remains unclear. Human studies have reported the presence of viral particles within the renal glomeruli post-COVID-19, suggesting their potential involvement in triggering pathological changes [20,32,33]. Furthermore, our previous studies using a murine model of COVID-19 (the MHV-1 infected mouse model as a highly relevant model for the study of SARS-CoV-2 infection and multi-organ injury) have demonstrated various pathological alterations tubular epithelial degeneration, proximal and distal tubular necrosis, tubular-vessel congestion, vacuolation, and hemorrhage in interstitial tissue in both acute and long-term post-infections. In addition, we also found edema, inflammation, macrophage, lymphocyte infiltration, and severe tubular necrosis in long-term post-infection [17,18,20].

In this study involving MHV-1 infected mice, we aimed to investigate the association between long-term kidney changes, particularly fibrogenesis, and factors involved in kidney injury, considering growth factors and growth factor receptors, pro-inflammatory and pro-fibrotic markers such as KIM-1, EGFR, and TGF-β. The presence of KIM-1 suggests a potential role for this molecule in long-term renal injury (fibrosis), as it is recognized as one of the components involved in the pathogenesis of chronic kidney diseases [33,34,35]. We further explored other molecules such as IL-18, NGAL, FGF-23, TNFR1, YKL-40, TLR-2, HIF-1α, and MMP7, which are involved in fibrosis development MHV-1 infection. Additionally, we assessed whether the inhibition of viral infection by a newly identified 15-amino-acid synthetic peptide, SPIKENET (SPK), could prevent or ameliorate kidney dysfunction.

## 2. Materials and Methods

### 2.1. Mice

We used 8-week-old female A/J mice weighing 22–24 grams each. These mice were purchased from Jackson Laboratories (Bar Harbor, ME, USA), and were kept in cages at the University of Miami Miller School of Medicine animal isolation facility. The animals were fed daily with a standard lab chow diet (Envigo 2918 irradiated, Teklad diet, Dublin, VA, USA) and provided with water ad libitum (autoclaved tap water). The study was performed according to the guidelines of the University of Miami Institutional Animal Care and Use Committee (IACUC protocol number 20-131 LF).

### 2.2. Viral (MHV-1) Inoculation and SPIKENET Treatment

MHV-1 was purchased from the American Type Culture Collection (ATCC, cat# VR 261, Manassas, VA, USA). Mice were split into (1) a healthy control group, (2) an MHV-1 virus-inoculated group, and (3) an MHV-1 virus-inoculated group that is treated with SPIKENET (SPK). MHV-1 viral inoculation was performed as previously described [18,19,21,36,37,38]. Briefly, groups 2 and 3 have been inoculated intranasally with 5000 PFU MHV-1 and were observed to ensure adequate inhalation of the virus. MHV-1 inoculated mice (group 3) were additionally treated with 5 mg/kg body weight of SPIKENET (SPK) as previously described by us [18,19,21,38].

### 2.3. Kidney Collection and Storage

At the end of the study period (7 days post-infection for acute studies and 12 months post-infection for long-term studies), kidneys were collected, and half of the kidneys were fixed in 10% formalin and the other half were stored in −80 °C for further analysis. Paraffin-embedded sections (processed through Histoscore PELORIS 3 Premium Tissue Processing System, Leica Biosystems Inc., Buffalo Grove, IL, USA), were then cut into 10 μm thick pieces by an ultra-thin semiautomatic microtome (Histoscore auto cut automated rotary microtome, Leica Biosystems Inc., Buffalo Grove, IL, USA) and immunofluorescence was performed for various proteins as detailed below.

### 2.4. Immunofluorescence

Paraffin-embedded tissue sections from healthy control and MHV-1 infected group (10 microns) were incubated with specific antibodies to EGFR (cat# 05-1047 monoclonal antibody, Millipore Sigma, Burlington, MA, USA); TLR-2 (cat# 06-1119 polyclonal antibody, Millipore Sigma); HIF-1α (cat# H1Alpha67, ab1 monoclonal antibody, Abcam, Cambridge, UK); KIM-1 (cat# A2831 polyclonal antibody, ABclonal, Woburn, MA, USA); YKL-40 (cat# A20792 polyclonal antibody, ABclonal); MMP-7 (cat# AF907 polyclonal antibody, R&D systems, Minneapolis, MN, USA). All were used at 1:500 dilution. The sections were then incubated with respective secondary antibodies (Alexa flour 488 or Alexa flour 546 goat anti-rat, mouse, or rabbit IgG (Life Technologies, San Diego, CA, USA) at a concentration of 1:250 to 1:500. Fluorescent images from these sections were randomly collected with Zeiss LSM510/UV Axiovert 200 M confocal microscope (Carl Zeiss Microscopy, LLC, Thornwood, NY, USA) and quantified using Velocity 6.0 High-Performance Cellular Imaging Software (PerkinElmer, Waltham, MA, USA) as described previously, and normalized to the number of DAPI-positive cells, as well as to the area and intensity of DAPI.

### 2.5. Immunoblot

Kidney tissues from healthy control and MHV-1-infected group were subjected to gel electrophoresis and immunoblotting as described previously [18,19,21,38]. Briefly, equal amounts of proteins from uninfected, infected, and drug-treated mice kidney tissue extracts were subjected to SDS–polyacrylamide gel electrophoresis and then electrophoretically transferred. The membranes were then incubated with primary antibodies to EGFR (cat# 05-1047 monoclonal antibody, Millipore Sigma, Burlington, MA, USA); TLR-2 (cat# 06-1119 polyclonal antibody, Millipore Sigma); HIF-1α (cat# H1Alpha67, ab1 monoclonal antibody, Abcam, Cambridge, UK); KIM-1 (cat# A2831 polyclonal antibody, ABclonal, Woburn, MA, USA); YKL-40 (cat# A20792 polyclonal antibody, ABclonal); MMP-7 (cat# AF907 polyclonal antibody, R&D systems, Minneapolis, MN, USA). All were used at 1:2,000 dilution. Horseradish peroxidase-conjugated anti-rabbit and anti-mouse secondary antibodies (Vector Laboratories, Newark, CA, USA) were used at 1:5000 dilution. The optical density of the bands was measured with the Sigma Scan Pro program (Jandell Scientific, CA, USA) as a proportion of the signal of a housekeeping protein band (β-actin) [38] (Table S1).

### 2.6. Real-Time PCR

Total RNA was extracted from the kidney tissues according to the manufacturer’s protocol using Qiagen RNeasy mini kit (Cat #74104, Qiagen, Hilden, Germany). Briefly, 10 mg of kidney tissue was homogenized using RLT (lysis buffer) buffer followed by RNA purification using a RNeasy spin column. Total RNA was determined based on the absorbance at 260 nm using a nano dropper (nanodrop, Fisher Scientific, Waltham, MA, USA). A total of 1 μg/sample was subjected to cDNA synthesis using a cDNA reverse transcription kit (Cat #11904-018, Invitrogen, Waltham, MA, USA) according to the manufacturer’s protocol. Specific mouse primers (see Table 1 for the list of primers) were designed using a primer bank and purchased from Oligos, Sigma Aldrich (Burlington, MA, USA). Quantitative real-time PCR was performed using an icycler (Bio-Rad Laboratories, Hercules, CA, USA) with the threshold cycle number determined by icycler software version 3. All the reactions were carried out in triplicates and the threshold values were calculated and normalized to the average CT value of β-actin. The data were analyzed by one-way ANOVA with Tukey’s multiple comparison test using GraphPad 9.5.1 software. *p* values less than 0.05 were considered statistically significant.

## 3. Results

### 3.1. Epidermal Growth Factor Receptor (EGFR)

**Figure 1:**
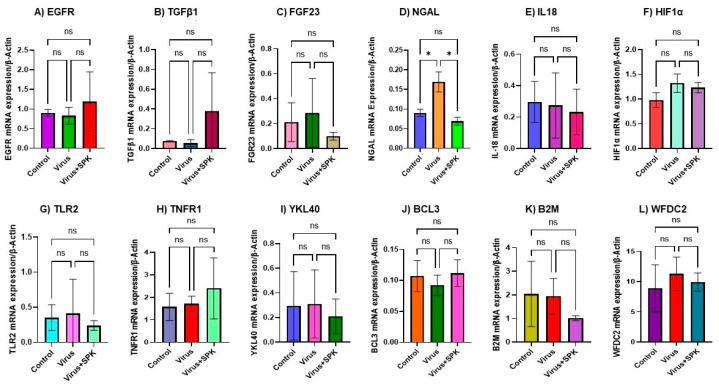
Altered mRNA expressions 7-days post-MHV-1 infection (acute). (**A**) EGFR mRNA level is not significantly changed in infected as well as treatment with SPK. (**B**) TGFβ mRNA level is not significantly changed in infected as well as treatment with SPK. (**C**) FGF23 mRNA level is not significantly changed in infected as well as treatment with SPK. (**D**) NGAL mRNA level is significantly increased in the infected group, while its mRNA level is significantly decreased following SPK treatment. (**E**–**L**) mRNA level is not significantly changed in the infected group, while its mRNA level decreased not significantly following SPK treatment. Values are the mean SD of three independent experiments. * = *p* < 0.05 is statistically significant. ns = nonsignificant.

**Figure 2:**
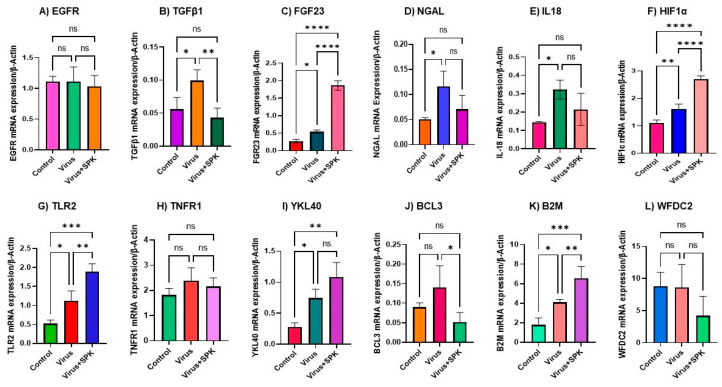
Altered mRNA expressions 12 months post-MHV-1 infection (chronic). (**A**) EGFR mRNA level is not significantly changed in infected as well as treatment with SPK. (**B**) TGF-β1 mRNA level is significantly increased in the infected group, while its mRNA level is significantly decreased following SPK treatment. (**C**) FGF-23 mRNA level is significantly increased in the infected group, and treatment with SPK leads to substantial elevation. (**D**) NGAL mRNA level is significantly increased in the infected group, while its mRNA level decreased not significantly following SPK treatment. (**E**) IL-18 mRNA level is significantly increased in the infected group, while its mRNA level decreased not significantly following SPK treatment. (**F**) HIF-1α mRNA level is significantly increased in the infected group, and treatment with SPK leads to substantial elevation. (**G**) TLR-2 mRNA level is significantly increased in the infected group, and treatment with SPK leads to significant elevation. (**H**) TNF-R1 mRNA level is not significantly changed in infected as well as treatment with SPK. (**I**) YKL-40 mRNA level is significantly increased in the infected group, while its mRNA level increased not significantly following SPK treatment. (**J**) BCL3 mRNA level is not significantly increased in the infected group, while its mRNA level is significantly decreased following SPK treatment. (**K**) B2M mRNA level is significantly increased in the infected group, and treatment with SPK leads to significant elevation. (**L**) WFDC2 mRNA level is not significantly changed in infected as well as treatment with SPK. Values are the mean SD of three independent experiments. * = *p* < 0.05 is statistically significant; ** = *p* < 0.01 is statistically significant; *** = *p* < 0.001 is statistically significant; **** = *p* < 0.0001 is statistically significant; ns = nonsignificant.

**Figure 3:**
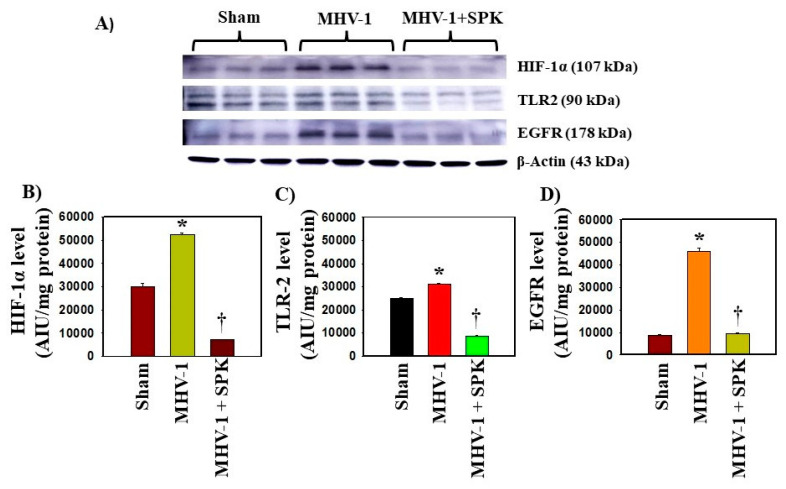
(**A**) Representative immunoblots changes in HIF1α, TLR2, and EGFR protein levels. All three protein levels increased 12 months post-infection in kidney cells in mice. Treatment with SPK (5 mg/kg) reduced all three protein levels significantly; HIF-1α and TLR2 levels are reduced below the normal (**B**,**C**) and EGFR levels, approximating the control (**D**). Values are the mean SD of three independent experiments. * *p* < 0.05 is statistically significant vs. Sham; † *p* < 0.05 is statistically significant vs. MHV-1.

**Figure 4:**
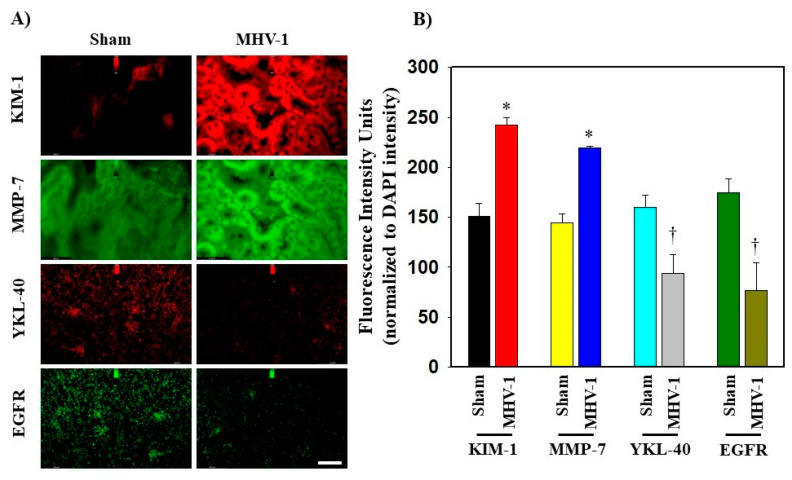
(**A**,**B**) Alteration of the immunofluorescence levels of KIM-1, MMP-7, YKL-40, and EGFR in the kidneys of mice 7 days post-MHV-1 infection. KIM-1 and MMP-7 levels are significantly elevated compared to the normal group (Sham). However, YKL-40 and EGFR expressions are markedly reduced in comparison with the health control (Sham). Values are the mean SD of three independent experiments. Scale bar = 35 mm. * *p* < 0.05 is statistically significant increase vs. Sham; † *p* < 0.05 is considered to be statistically significant decrease vs. MHV-1.

**Figure 5:**
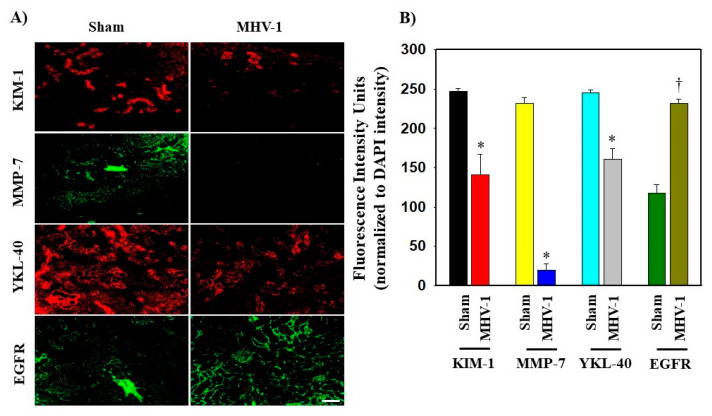
(**A**,**B**) Alteration of the immunofluorescence levels of KIM-1, MMP-7, YKL-40, and EGFR in the kidneys of mice 12 months post-MHV-1 infection. KIM-1, MMP-7 and YKL-40 levels are significantly reduced compared to the normal (Sham). However, EGFR expressions are increased in comparison with the health control (Sham). Fluorescence intensities for those markers confirmed the immunofluorescence findings. Values are the mean SD of three independent experiments. Scale bar = 35 mm. * *p* < 0.05 is a statistically significant decrease vs. Sham; † *p* < 0.05 is considered to be a statistically significant increase vs. MHV-1.

Certain growth factors and their corresponding cellular signaling pathways have been implicated in the pathogenesis and development of fibrosis in various organs [39,40,41]. Pulmonary fibrosis has been reported in individuals infected with SARS-CoV, and this fibrotic process is mediated by the EGFR signaling pathway [40]. Since EGFR stimulation is involved in renal fibrosis in other conditions [41], and our histopathological observations indicate severe and irreversible kidney dysfunction in the long-term post-infection period [21], we investigated whether EGFR stimulation occurs in kidneys following acute and long-term post-SARS-CoV-2 infection using a surrogate mouse model. Additionally, we aimed to determine if this event is involved in the development of kidney fibrosis. We found no significant change in the mRNA expression of EGFR in the kidneys following acute or long-term post-infection (Figure 1A and Figure 2A). Further, treatment of these mice with SPIKENET (SPK) (5 mg/kg), a synthetic peptide that prevents the binding of spike protein to its respective receptor CEACAM1, did not alter EGFR mRNA expression in these kidneys (Figure 1A and Figure 2A). To further investigate EGFR alterations, we performed an immunoblot analysis to examine EGFR protein levels. This analysis revealed a notable four-fold increase in EGFR protein levels in long-term post-MHV-1 infection, as compared to healthy controls (Figure 3A) (see supplementary material for whole immunoblots Figures S1–S4). Additionally, the administration of SPK (5 mg/kg) almost normalized the EGFR protein level, reducing it 3.8-fold compared to MHV-1 infected mice (Figure 3D). Moreover, immunofluorescence investigation found a significant reduction (42%) in the EGFR expression in the kidney tissue of acutely infected mice (Figure 4A,B). However, the EGFR level was significantly increased (52%) in the long-term post-infected group (Figure 5A,B).

### 3.2. Transforming Growth Factor-β (TGF-β)

TGF-β is widely known as a principal contributor to the development and progression of fibrosis across various organs. Its role primarily involves the activation of canonical and non-canonical signaling pathways which subsequently elicit extracellular matrix (ECM) deposition [41,42]. In our results, we found no significant changes in the mRNA levels of TGF-β1 within the kidneys of acutely infected mice (7 days) or those treated with SPK (Figure 1B). Conversely, we found a significant increase in TGF-β mRNA levels in kidneys 12 months post-MHV-1 infection (Figure 2B). These findings strongly suggest that the initiation of the fibrotic process may occur in the kidneys of long COVID since treatment of these infected mice with SPK resulted in a significant reduction of TGF-β mRNA levels (Figure 2B).

### 3.3. Fibroblast Growth Factor 23 (FGF23)

FGF23 expression is known to increase in response to inflammation and hypoxia, ultimately leading to chronic kidney disease (CKD) [43]. Notably, COVID-19-infected patients with a history of CKD exhibited elevated levels of FGF23 [44]. Our results demonstrate that there was no significant change in the mRNA expression of FGF-23 within the acutely infected group, as well as in the group treated with SPK (Figure 1C). In the long-term post-infection group, however, we observed a significant increase in FGF23 expression, which was further exacerbated by treatment with SPK (5 mg/kg) (Figure 2C).

### 3.4. Kidney Tubular Markers

Kidney injury molecule-1 (KIM-1), neutrophil gelatinase-associated lipocalin (NGAL), and matrix metalloproteinase-7 (MMP-7) have been implicated in the pathogenesis of acute and long-term post-kidney injury [33,45,46,47].

#### 3.4.1. Kidney Injury Molecule-1 (KIM-1)

In our study, immunofluorescence examination showed a significant 39% increase in KIM-1 levels within the acutely infected group compared to the sham group (control) (Figure 4A,B). Conversely, a significant reduction in KIM-1 level (43%) was observed in the long-term post-infection group (Figure 5A,B).

#### 3.4.2. Neutrophil Gelatinase-Associated Lipocalin (NGAL)

Additionally, we found a significant increase in NGAL mRNA expression in acutely and chronically infected mice compared to the control (Figure 1D and Figure 2D). Notably, treatment of the acutely infected mice with SPK (5 mg/kg) resulted in a significant reduction in NGAL mRNA expression (Figure 1D). However, in the chronically infected counterpart, no significant reduction in NGAL mRNA expression was observed (Figure 2D).

#### 3.4.3. Matrix Metalloproteinase-7 (MMP-7)

Regarding MMP-7, the immunofluorescence analysis revealed an increased protein level in acutely infected mice (Figure 4A,B) whereas in the chronically infected group, the protein level was reduced, as compared to sham (Figure 5A,B). These results confirm the significance of ameliorating damage and promoting renal tubular regeneration following acute kidney injury (AKI). However, the decreased expression of MMP-7 in CKD is associated with fibrosis, suggesting the loss of the protective function observed in AKI [47].

### 3.5. Kidney Inflammatory Markers

#### 3.5.1. Interleukin 18 (IL-18)

The direct infection of the kidney by the SARS-CoV-2 virus results in an inflammatory response characterized by the release of cytokines and interleukins [48]. Among these cytokines, elevated levels of IL-18 have been observed in severe AKI [48]. In our mice model, we investigated the mRNA expression of IL-18, and our results indicate no significant changes within the acutely infected group (Figure 1E). However, in the long-term post-infected group, we observed a significant 55% increase in IL-18 mRNA levels (Figure 2E). There was no significant reduction in IL-18 mRNA expression observed in either the acutely or chronically infected groups treated with SPK (Figure 1E and Figure 2E).

#### 3.5.2. Hypoxia-Inducible Factor 1α (HIF-1α)

SARS-CoV-2-mediated hypoxia induces the activation of multiple genes, including HIF-1α, which is involved in fibrogenesis [49]. Our study revealed no significant alterations in HIF-1α mRNA expression during acute infection (Figure 1F). However, in the context of long-term infection, we observed a significant increase in both mRNA expression (Figure 2F) and protein levels of HIF-1α (Figure 3B) compared to control. In the chronically infected group treated with SPK, we observed a substantial elevation in mRNA expression (Figure 2F), but Western blot analysis demonstrated a significant reduction in protein level (Figure 3A,B). While we do not know the reason for such a change, it is possible that since HIF-1α production is effectively controlled by post-transcriptional mechanisms likely due to RNA-binding proteins (RBPs) and the non-coding RNAs that interact with the HIF-1α mRNA; this may have influenced its half-life and translation rate.

#### 3.5.3. Toll-like Receptors

TLR-2 and TLR-4 upregulation has been documented in CKD [50]. Within the acutely infected group, we found no significant changes in TLR-2 mRNA expression (Figure 1G). However, in the long-term post-infected group, a significant increase in TLR-2 mRNA expression was observed (Figure 2G). This result was further confirmed by Western blot analysis (Figure 3A,C). To explore the impact of our experimental drug, SPK, on TLR2 mRNA levels in both the acutely and chronically infected groups, we conducted additional investigations. Interestingly, we observed no changes in TLR-2 mRNA expression in the acutely infected group following treatment with SPK (Figure 1G). However, in the chronically infected group treated with SPK, a further increase in TLR-2 mRNA expression was observed (Figure 2G). On the other hand, Western blot analysis demonstrated a significant reduction of TLR-2 protein levels in chronic MHV-1-infected mice treated with SPK (Figure 3A,C).

#### 3.5.4. Tumor Necrosis Factor Receptor 1 (TNFR1)

Tumor necrosis factor alpha (TNFα) and its receptors, TNFR1 and TNFR2, have been shown to be elevated in renal diseases associated with SARS-CoV-2 infection across diverse patient populations [51]. Surprisingly, we observed no significant difference in the expression of TNFR1 mRNA among the acutely and chronically MHV-1-infected mice, as well as in the SPK-treated mice, when compared to controls. (Figure 1H and Figure 2H).

#### 3.5.5. Chitinase-4-like Protein 1 (YLK-40)

YKL-40 has emerged as a novel prognostic marker for severe SARS-CoV-2 infection [52]. Within the acute MHV-1-infected mice, we did not observe any significant alterations in YKL-40 mRNA expression compared to the control group (Figure 1I). Additionally, treatment with SPK did not result in significant changes in YKL-40 mRNA expression (Figure 1I). However, in the long-term infected group, we noted a substantial increase in YKL-40 mRNA expression, and treatment with SPK (5mg/kg) further augmented its mRNA expression compared to control (Figure 2I). Conversely, the immunofluorescence analysis showed a significant reduction of YKL40 levels by 44% in both acute and chronic MHV-1 infection groups compared to sham (Figure 4 and Figure 5). Notably, the elevated YKL40 mRNA observed in chronic MHV-1 infection may undergo degradation, leading to its reduced visualization in the immunofluorescence analysis.

#### 3.5.6. B-Cell Leukemia Protein 3 (BCL3)

BCL3 serves as an important regulator of nuclear factor kappa-light-chain-enhancer of activated B cells (NF-kB) and has been implicated in renal disease, exhibiting increased expression [53]. We found no significant change in BCL3 mRNA expression within the acute and chronic MHV-1 infected groups (Figure 1J and Figure 2J). Furthermore, treatment of acutely infected mice with SPK (5 mg/kg) did not result in significant alterations in BCL3 mRNA expression (Figure 1J). However, we have found a significant reduction of BCL3 mRNA expression in the long-term infected group receiving SPK (5 mg/kg) treatment (Figure 2J).

#### 3.5.7. Beta-2 Microglobulin (B2M)

B2M is a protein secreted by all nucleated cells at a constant rate, filtered by the glomerulus, and subsequently reabsorbed by proximal tubular cells. Elevated levels of B2M in serum and urine are used to assess kidney function in AKI and CKD [54]. We did not find any significant change in B2M mRNA expression within the acute MHV-1-infected group or its SPK-treated counterpart (Figure 1K). However, in the context of chronic MHV-1 infection, we found a significant increase in the B2M mRNA expression (Figure 2K), which was further augmented by SPK (5 mg/kg) treatment (Figure 2K).

#### 3.5.8. WAP Four-Disulfide Core Domain 2 (WFDC2)

WFDC2 has emerged as a promising clinical biomarker for kidney disease and fibrosis [55]. In our study, we found no significant change in the mRNA expression of WFDC2 in either the acute or chronic MHV-1 infected groups, as well as the SPK treatment groups, when compared to controls (Figure 1L and Figure 2L).

### 3.6. Results Summary

In summary, our study revealed the involvement of several key proteins in the development of kidney fibrosis following both acute and chronic MHV-1 infection. These proteins include TGF-β1, HIF-1α, NGAL, B2M, FGF23, TLR2, IL-18, EGFR, YKL-40, KIM-1, and MMP7. A summary of the mRNA expression changes observed in these proteins is presented in Table 2, and the protein level changes for both immunoblot and immunofluorescence studies are presented in Table 3 and Table 4, respectively.

## 4. Discussion

Our study demonstrates the possible development of renal fibrosis in long-term post-infection for the first time. In particular, we found increased TGF-β1, FGF23, NGAL, IL-18, HIF1-α, TLR2, YKL-40, and B2M mRNA levels long-term post MHV-1 infection. Immunoblot studies showed an elevation in protein levels of HIF1-α, TLR-2, and EGFR long-term post-MHV-1 infection. However, only KIM-1 and MMP-7 protein levels and NGAL mRNA are increased in acute infection (7 days). Moreover, treatment of MHV-1 infected mice with a synthetic peptide, SPIKENET (SPK), which inhibits spike protein binding, reduced NGAL mRNA in acute infection, and decreased TGF-β1, BCL3 mRNA, and EGFR, HIF1-α, and TLR-2 protein levels long-term post MHV-1 infection. These findings strongly suggest the possible development of kidney fibrosis in the long-term post-infection. Therefore, targeting these factors may pave the path to preventing long-term complications with COVID-19.

The main hypothesis of this study is to investigate the pathophysiology of MHV-1 (coronavirus) on short and long-term kidney injuries through analysis of different fibrogenesis molecules. Those molecules can participate via direct and/or indirect pathways in renal injury and fibrosis. Therefore, for the first time, we conducted a comprehensive renal investigation, spanning both short-term (7 days) and long-term (12 months) durations, to analyze the involvement of various molecules in kidney fibrosis of mice infected with MHV-1. We found that a majority of those molecules exhibited increased levels in cases of chronic infection, as compared to the sham group (Table 2). Previously, we reported the role of SPK in the prevention of viral entry and replication in the kidney, liver, and heart [19]. Consequently, we investigated the effect of SPK on renal fibrogenesis molecules.

Long-term complications arising from SARS-CoV-2 infection have been observed in various patients and studied across multiple organs including the heart, lungs, brain, etc. Some of these complications, initially manifesting during the acute phase of infection, exhibit a persistent nature. SARS-CoV-2 impacts the renal through direct invasion of distinct kidney cells or indirectly via immune-mediated cytokine storm impacts renal function and contributes to the development of kidney fibrosis [20,33,34].

Several studies demonstrated the effects of COVID-19 infection on kidneys in humans. Large observational analyses in USA, Brazil, and Europe determined a high incidence of COVID-19 infection and AKI (34%) [1,3]. The studies showed that AKI was diagnosed in hospitalized patients with different stages including stage I (66%), II (20%), and III (35%) [1,4,5]. Further, the authors mentioned that stage III was observed more in the patients who were admitted to the intensive care unit [1,4,5]. Observational analyses that occurred in Spain and UK showed COVID-19 patients are more prone to develop AKI during hospitalization and progress to chronic kidney disease [1,4,5,10]. An investigational study performed by Kudose et al. on 19 patients infected with SARS-CoV-2 who developed AKI showed different pathological changes among those patients [8]. The author found a wide spectrum of glomerular and tubular diseases including minimal change disease, lupus nephritis, membranous glomerulopathy, anti-glomerular basement membrane, and focal segmental glomerulosclerosis. Pathophysiology is complex and involves multiple factors that initiate the AKI and progressively lead to chronic kidney disease (CKD) [3,4,5,8,9,10,56].

Multiple studies have substantiated the involvement of EGFR in the development of organ fibrogenesis [40,57]. Activation of EGFR by its ligand Epiregulin establishes a positive feedback loop through the neurogenic locus notch homolog protein (NOTCH) signaling pathway [58]. Moreover, EGFR overexpression plays a role in the activation of TGF-β-mediated renal fibrosis, whereas inhibition of EGFR effectively mitigates TGF-β-induced renal fibrosis [41]. In our study, the elevation of EGFR protein levels in the chronically infected group suggests its collaboration with various factors and pathways to induce renal fibrosis (needs further investigation). Notably, we found a remarkable efficacy of SPK in reducing EGFR protein levels, which holds promising potential in preventing fibrosis.

TGF-β is a pivotal factor in numerous inflammatory processes and fibrosis. The involvement of TGF-β in fibrogenesis is intricate, as it stimulates the matrix-preserving transcriptional program in fibroblasts. This activation occurs through both SMAD-dependent pathways, involving type II β receptor-activin receptor-like kinase 1/5-SMAD2/3 axis, as well as non-SMAD pathways mediated by endoglin, beta-glycan, BMP, and activin membrane-bound inhibitor homolog (BAMBI) [59,60,61]. In our study, we observed an elevation in TGF-β mRNA levels in cases of chronic MHV-1 infection, aligning with the increased TGF-β levels reported in other diseases associated with fibrogenesis. Additionally, the subsequent reduction of TGF-β expression following treatment with SPK highlights the effectiveness of this medication in attenuating fibrogenesis and the potential development of CKD in long SARS-CoV-2 infection.

FGF23 promotes the profibrotic signaling pathways in renal fibroblasts in AKI, leading to renal fibrosis and CKD [62,63]. The increased expression of the FGF23 gene observed in the long-term MHV-1 infection mice suggests its direct or indirect contribution to renal fibrosis in chronically infected individuals. Notably, our findings demonstrate a further elevation in the FGF23 mRNA levels following SPK treatment.

BCL-3 (a proto-oncogene) acts as a regulator of NF-κB and is inducible during kidney injury in response to the inflammatory response. Specifically, BCL3 expression is observed to be increased in the tubular epithelium following stimulation by pro-inflammatory cytokines [53,64]. Chen et al. reported a significant elevation of BCL-3 mRNA levels in mice with chronic kidney disease, unrelated to SARS-CoV-2 infection [64]. In our study, we did not find any significant increase in BCL3 expression in the chronic MHV-1 group. However, following treatment with SPK, the expression of BCL3 was significantly reduced. These findings suggest that SPK administration may effectively slow the progression of renal fibrosis by modulating BCL3 expression.

B2M was previously known to be involved in renal injuries [54]. In our study, we found significantly increased B2M expression in mice with chronic MHV-1 infection. The finding aligns well with previous reports and provides further confirmation of the role of B2M in tubular injury-mediated fibrosis.

WFDC2, also referred to as HE4 is a protein primarily found in the reproductive tract. Its elevated levels have been linked with ovarian, breast, and lung adenocarcinoma [65]. Moreover, WFDC2 is expressed in epithelial cells of normal tissue and is involved in innate immunity. Recently, WFDC2 has been utilized as a marker for monitoring the severity of SARS-CoV-2 infection, ranging from mild to severe cases [65]. However, Chen et al. observed an elevation of WFDC2 in mice kidney cells, which has recently been identified as a marker of renal fibrosis [65]. Noteworthy, the progression of CKD in SARS-CoV-2 infection is related to fibrogenesis that is triggered via indirect activation of WFDC2 through BCL-3 upregulation. However, no significant change was found in both acute and chronic MHV-1 infection and treatment groups.

SARS-CoV-2 triggered inflammation is thought to stimulate nod-like receptor protein (NLRP3)-inflammasome, resulting in significant production of IL-18 [66]. IL-18 is recognized as a proinflammatory cytokine that, in conjunction with interleukin 12 (IL-12), promotes the release of interferon-gamma (IFN-γ) and induces a robust T-helper-1 response [67,68]. Further, interleukin 18 (IL-18) stimulates myeloid differentiation factor 88 (MyD88)/nuclear factor kappa B (NF-κB), as well as increases the production of interleukin 4 (IL-4) and interleukin 13 (IL-13), which stimulate T-helper-2 cell. IL-18 has been identified as a potential biomarker for renal disorders in patients with and without SARS-CoV-2 infection [67,68]. In our study, we observed a significant increase in IL-18 expression in the long-term post-infected group. These findings suggest the activation of various inflammatory signaling pathways that contributed to the progression of renal fibrogenesis in SARS-CoV-2 infection. Although SPK treatment did not demonstrate a significant reduction in IL-18 expression, it plays an important role in slowing or halting renal fibrogenesis by modulating other factors within the NF-κB pathway during the development of fibrosis.

Chronic hypoxia has been recognized as a driver of kidney fibrosis [69]. HIF-1α is deemed to be a major mediator of the hypoxic response and plays a key role in the development of renal fibrosis in mice [70,71]. In the case of SARS-CoV-2 infection, HIF-1α can be stimulated through different pathways, including direct gene activation, mitochondrial damage, and cytokine production. Elevated levels of HIF-1α have been associated with increased mortality and induction of fibrogenesis through stimulation of excessive extracellular matrix deposition, or other pathways mentioned above. In our investigational analysis, we found a significant increase in HIF-1α mRNA expression and protein levels in the long-term post-infection group. These findings correlate with the previous studies on renal fibrosis with different diseases other than SARS-CoV-2 infection. We administered SPK to the chronically infected animals and observed a notable reduction in HIF-1α protein levels. This suggests that SPK has the potential to prevent further renal fibrosis in the context of SARS-CoV-2 infection.

We investigated TLR2 in our study, given its extensive involvement in various pathways that contribute to pathological changes in various organs. TLR2, along with TLR4, interacts with high mobility group box 1 (HMGB-1) to activate the NF-κB signaling pathway which, in turn, stimulates the production of proinflammatory cytokines such as IL-1β, TNF-α, and IFN-γ [72]. These cytokines further activate macrophages, thereby promoting organ fibrogenesis, particularly in the kidney [72]. In an animal study, Khan et al. found that SARS-CoV-2 spike proteins activate TLR2 [73]. In our investigation, we confirmed the elevation of TLR2 mRNA levels in long-term post-infection through Western blotting analysis. This suggests that acute infection with COVID-19 can trigger a persistent and sustained activation of TLR2, thereby enhancing fibrogenesis in the kidneys. Interestingly, utilization of SPK leads to the reduction of TLR2 protein levels. This outcome suggests that early treatment of infected individuals with COVID-19 by SPK can inhibit/slow the development of renal fibrosis.

TNFR1 and TNFR2 are cytokine receptors responsible for the binding and activation of TNF-α during inflammation and tissue injury [74]. Specifically, TNFR1 is ubiquitously expressed in all cells of the lymphoid system, including glomerular and peritubular endothelial cells. Activation of the NF-kB pathway occurs through the formation of TNF-α/TNFR1 complexes [75,76,77]. Mortaz E. et al. reported a connection between the severity of COVID-19 and elevated levels of soluble TNFR1 in the plasma of COVID-19 patients [7]. However, in our study using the MHV-1 mouse model of COVID-19, we found no significant changes in TNFR1 expression in both acute and chronic MHV-1 infection and treatment groups. Hence, further studies are needed to identify the role of TNFR1 in the development of renal fibrosis.

YKL-40 is a 40-kDa glycoprotein belonging to the chitinase-like protein family. It is produced by inflammatory cells, including macrophages and neutrophils, as well as malignant cells, and serves to regulate vascular endothelial growth factor (VEGF). Additionally, YKL-40 plays a role in inflammation, angiogenesis, extracellular matrix production, fibrogenesis, atherosclerosis, and endothelial dysfunction [78]. Through its interaction with TGF-β, YKL-40 can drive the inflammatory pathways, inhibit apoptosis, and initiate fibrogenesis [79]. Moreover, YKL-40 is essential for stimulating alternative macrophage activation [78]. In patients with hemodialysis, elevated levels of YKL-40 have been associated with increased mortality [78]. To date, there have been no studies specifically targeting YKL-40 in the context of COVID-19-related renal fibrosis. In our investigation, we found no significant increase in YKL-40 mRNA expression in mice during the acute phase of infection. However, in the long-term post-infected group, there was a significant increase in YKL-40 expression. Conversely, immunofluorescence analyses in both acute and chronic infection groups revealed a substantial reduction of YKL40 expression compared with control. It is important to note that the elevation of YKL40 mRNA levels may be attributed to either the degradation of YKL40 molecules or the activation of an alternative pathway that is unresponsive to treatment, and therefore SPK did not reduce the YKL40 mRNA levels.

Plasma and serum NGAL have been identified as promising early predictive biomarkers for the development of AKI compared to traditional creatinine [45]. In our study, we observed elevated levels of NGAL in acute MHV-1 infection, which is indicative of acute tubular injury accompanied by inflammation. Komaru Y et al. also reported the correlation between elevated urinary NGAL and AKI in COVID-19 patients [80]. These findings align with previous reports highlighting NGAL as a reliable marker for AKI. Additionally, NGAL has shown promise as a marker for predicting severe kidney diseases, surpassing the performance of cystatin C [45]. We also noted an increase in the expression of NGAL expression in chronic MHV-1 infection. These findings suggest that the elevated levels of NGAL may be a potential marker for AKI, fibrosis, and CKD in the setting of SARS-CoV-2-mediated acute and chronic infection. Further, it is important to note that SPK inhibited the NGAL expression only in acute infection, but not long-term post-infection. While the reason for such differential response of SPK in acute and long-term post-infection is unclear, it is possible that the short-term SPK treatment may not be enough to inhibit the NGAL in the long term.

Numerous investigations have revealed the involvement of KIM-1 in the regulation of viral infection, autoimmune diseases, and atopic disorders [81,82,83,84,85]. Generally, KIM-1 levels in renal tissue remain low, but under conditions of renal pathological changes, its concentration can rise significantly. This elevation further triggers various pathways involved in the development of renal fibrosis. The rise of KIM-1 within the kidney stimulates extracellular signal-regulated kinase (ERK1/2) and signal transducer and activator of transcription 3 (STAT3) [81,82,83,84,85]. Additionally, KIM-1 binds to STAT3, leading to an augmentation of its mRNA and 77 levels [81,82,83,84,85]. This, in turn, provokes a heightened immune cell response, severe inflammation, and upregulation of HIF-1α levels, all of which contribute to acute and chronic kidney disorders [81,82,83,84,85]. Notably, KIM-1 is recognized as a diagnostic marker for acute tubular necrosis of the kidney (approved by the US Food and Drug Administration) [81,82,83,84,85]. In our immunofluorescence analysis, we observed a significant increase in KIM-1 levels during acute infection. Hypothetically, the elevated level of KIM-1 in the acute setting may activate or trigger different pathways (this needs further investigation) to induce renal fibrogenesis. Following this activation, the levels of KIM-1 tend to decrease long-term post-infection.

Normally, the level of MMP-7 in kidney tissue is very low, making it a favorable prognostic biomarker for acute and chronic renal disorders [86,87]. The function of MMP-7 in renal disorders is multifaceted: it can exhibit protective function in acute kidney injury by degrading E-cadherin and Fas-Ligand (a TNF family protein) [86,87]. However, MMP-7 can also contribute to the progression of fibrosis and CKD by breaking down E-cadherin, leading to the liberation and nuclear translocation of β-catenin [86,87]. Furthermore, MMP-7 promotes the degradation of nephrin, which compromises the integrity of the slit diaphragm and ultimately leads to proteinuria and renal fibrosis [86,87]. Animal studies have demonstrated that TGF-β enhances the expression of MMP-7, and both factors play a role in triggering fibrogenesis in the kidney [86,87]. In our immunofluorescence analysis, we observed a significant elevation in MMP-7 levels of acutely infected mice, contrasting the reduced levels observed in those mice long-term post-infection. These results strongly suggest that the presence of MMP-7 is important after AKI to mitigate injury in the context of SARS-CoV-2 infection and promote renal tubular regeneration. However, during the long-term post-infection phase, the correlation with renal fibrosis suggests the loss of the protective function observed during AKI. These findings will be helpful to identify the development of renal fibrosis and the progression of CKD in patients with long COVID and kidney injury.

From what is already known about organ fibrosis, once organ fibrosis has occurred the physiology of the organ will be permanently affected. Further, various markers that we studied, TGF-β, NGAL, IL-18, HIF-1α, TLR2, YKL-40, and B2M, have been shown to be involved in organ fibrosis leading to organ dysfunction. Increased serum and plasma NGAL were identified as a marker of CKD.

Our newly invented drug (a 15-amino-acid synthetic peptide, known as SPIKENET) was designed to prevent the binding of spike glycoproteins with their receptor(s), angiotensin-converting enzyme 2 (ACE2), and carcinoembryonic antigen-related cell adhesion molecule 1 (CEACAM1) (SARS-CoV-2 and MHV-1, respectively), ameliorated animal death and reversed altered levels of various changes, protein levels and oxidative stress post-MHV-1 infection [18], although one or more of the RNA levels are potentiated by SPK. While the reason for such changes is unclear, it is possible that SPK treatment may not affect the transcriptional activity of those genes.

According to the results we observed in this study, we highlighted the significant involvement of a wide spectrum of fibrogenesis factors in coronavirus infection-related kidney injuries (acute and chronic). We investigated all the possible fibrogenesis factors in this study to demonstrate the connection of those factors in the development of renal injuries. Several studies showed COVID-19 is a leading cause of renal injuries [3,4,5,8,9,10,57]. Therefore, SARS-CoV-2 has been shown to trigger various pathological changes in the glomeruli-tubular structures. Further, the potential pathophysiology of renal injury in long COVID had not been studied before this experiment. Now, we elaborate on the involvement of SARS-CoV-2 in renal fibrosis. Additionally, our medication (SPK) showed important molecular, clinical, and pathological improvement when used in the infected mice, opening the door for possible halting, preventing, or treating renal injury or fibrosis in humans.

While the MHV-1 mice model provides an opportunity to study long COVID, there are limitations (e.g., limited sample size since most of the animals die during the acute stage post-infection within 7–12 days, differences in viral receptor binding protein, etc.). However, it should be emphasized that, so far, there is no suitable experimental model to study long COVID, since the severity of death is significant in other animal models, in particular, in the humanized mouse models of SARS-CoV-2 infection limit the use of those models to study long COVID (e.g., the severity of death is significant in hamster models of SARS-CoV-2 infection, as compared to humans admitted in hospital due to severe SARS-CoV-2 infection). Further, while the NIH recommends the use of these transgenic mice (e.g., the use of the Syrian hamster model to study mechanisms and consequences of COVID-19), there were no histopathological changes in samples of inoculated or contact hamster brains, livers, hearts, or kidneys. This is a limitation of the hamster or other transgenic/humanized SARS-CoV-2 models as it would not be useful to study the extrapulmonary pathologies observed in patients with COVID-19. We, therefore, believe that our comprehensive pathological and functional changes that are identified after acute and long-term post-infection which are highly comparable to humans with acute and long-term post-SARS-CoV-2 infection outweigh the differences (e.g., concerns regarding the viral binding mechanisms, with CEACAM-1 in MHV-1 instead of ACE2 in SARS-CoV-2). It is possible that human blood/serum samples or post-mortem can be used to analyze the markers for long COVID, though this will not provide mechanistic information on the disease pathogenesis.

## 5. Conclusions

In summary, our findings suggest that a diverse group of factors is likely involved in the development of renal fibrosis in long COVID. These include growth factors, inflammatory, and tubular markers. Specifically, KIM1 and MMP7 protein levels are significantly increased in acute MHV-1 infections, while a significant increase in EGFR protein level was identified long-term post-infection. Regarding the mRNA expression, a significant increase in TGF-β1, HIF-1α, NGAL, B2M, FGF23, TLR-2, IL-18, and YKL-40 was observed in the long-term post-MHV-1 infection, whereas only NGAL was found to be significantly increased in the acute stage post-infection. Additionally, treatment of mice with SPK attenuated both mRNA expression of NGAL and TGF-β and protein levels of EFGR, HIF-1α, and TLR2. From this, it was observed that the SPIKENET abrogates the development of kidney fibrosis by directly affecting the SARS-CoV-2 entry. Collectively, these findings suggest that both EGFR and NGAL may be critical factors in initiating and developing long-term kidney injury in COVID-19. Therefore, targeting EGFR and NGAL could provide a therapeutic approach to preventing renal fibrosis in long COVID. However, further studies are needed to identify the role of NGAL and other markers in the fibrosis and progression of CKD to use in clinical trials.

## Figures and Tables

**Table 1:** Primers used in the qRT-PCR analysis.

Gene Name	Primer Sequence
EGFR	GCCATCTGGGCCAAAGATACC GTCTTCGCATGAATAGGCCAAT
HIF-1α	GTCCCAGCTACGAAGTTACAGC AGTGCAGGATACACAAGGTTT
TLR2	GCAAACGCTGTTCTGCTCAG AGGCGTCTCCCTCTATTGTATT
YKL40	GTACAAGCTGGTCTGCTACTTC ATGTGCTAAGCATGTTGTCGC
NGAL	TCTCAGTGCCGCCGATTACTA CTTGACAGTGAACACGATCTCA
B2M	GGCCCATCTTGCATTCTAGGG CAGGCAACGGCTCTATATTGAAG
TGFB1	ATGTCACGGTTAGGGGCTC GGCTTGCATACTGTGCTGTATAG
TNFR1	CCGGGAGAAGAGGGATAGCTT TCGGACAGTCACTCACCAAGT
BCL3	CCGGAGGCCCTTTACTACCA GGAGTAGGGGTGAGTAGGCAG
IL-18	CCTACTTCAGCATCCTCTACTGG AGGGTTTCTTGAGAAGGGGAC
WFDC2	CCAGAACTGCACGCAAGA CAGGAACCCTCCTTATCATTGG
FGF23	CAGGTGATGAGCAGAAGAT CAGTTCTCCGGGTCGAAATAG
β-Actin	ATGACCCAAGCCGAGAAGG CGGCCAAGTCTTAGAGTTGTTG

EGFR—epidermal growth factor receptor; HIF-1α—hypoxia-inducible factor 1 subunit alpha; TLR2—Toll-like receptor 2; YKL40—chitinase 3 like protein 1; NGAL—neutrophil gelatinase-associated lipocalin; B2M—A beta-2 microglobulin; TGFB1—transforming growth factor beta-1; TNFR1—tumor necrosis factor-alpha receptor-1; BCL3—B-cell lymphoma 3; IL-18—interleukin 18; WFDC2—WAP four-disulfide core domain protein 2; FGF23—fibroblast growth factor 23.

**Table 2:** Kidney factors’ mRNA expression.

Factors	mRNA Expression			
	Acute Infection (7 Days)	SPK Treatment in Acute Infection	Long-Term Infection (12 Months)	Long-Term Post-SPK Treatment
EGFR	—	—	—	—
TGF-β1	—	—	↑	↓↓
FGF23	—	—	↑	↑↑↑↑
NGAL	↑	↓	↑	—
IL-18	—	—	↑	—
HIF-1α	—	—	↑↑	↑↑↑↑
TLR2	—	—	↑	↑↑
TNF-R1	—	—	—	—
YKL-40	—	—	↑	—
BCL3	—	—	—	↓
B2M	—	—	↑	↑↑
WFDC2	—	—	—	—

↑ = significant increase; ↓ = significant decrease; — = no significant changes.

**Table 3:** Immunoblots analysis.

Proteins	Protein Level	
	Long-Term Infection (12 Months)	SPK Treatment
EGFR	↑	↓
HIF-1α	↑	↓
TLR2	↑	↓

↑ = significant increase; ↓ = significant decrease.

**Table 4:** Immunofluorescence examination.

Proteins	Protein Level	
	Acute Infection (7 Days)	Long-Term Post-Infection (12 Months)
EGFR	↓	↑
YKL-40	↓	↓
KIM-1	↑	↓
MMP7	↑	↓

↑ = significant increase; ↓ = significant decrease.

## Data Availability

The data presented in this study are available on request from the corresponding author. The data are not publicly available due to the University of Miami Miller School of Medicine’s privacy policy.

## Supplementary Materials

The following are available online at https://www.mdpi.com/article/10.3390/biology12081070/s1, Figure S1: Western blot membrane of HIF-1α protein detected with anti- HIF-1α antibody; Figure S2: Western blot membrane of TLR2 protein detected with anti-TLR2 antibody; Figure S3: Western blot membrane of EGFR protein detected with anti-EGFR antibody; Figure S4: Western blot membrane of β-Actin protein detected with anti-β-Actin antibody; Table S1: densitometry data of HIF-1α, TLR2 and EGFR from PVDF membrane.
