# Supplementary material for: Kidney Damage in Long COVID: Studies in Experimental Mice
Source: Biology (Basel). 2023 Jul 30;12(8):1070. doi: 10.3390/biology12081070 (PMC10452084; doi:10.3390/biology12081070)
Supplement: Supplementary file 1 [file biology-12-01070-s001.zip › biology-2506374-supplementary-1.pdf]

Supplementary Materials

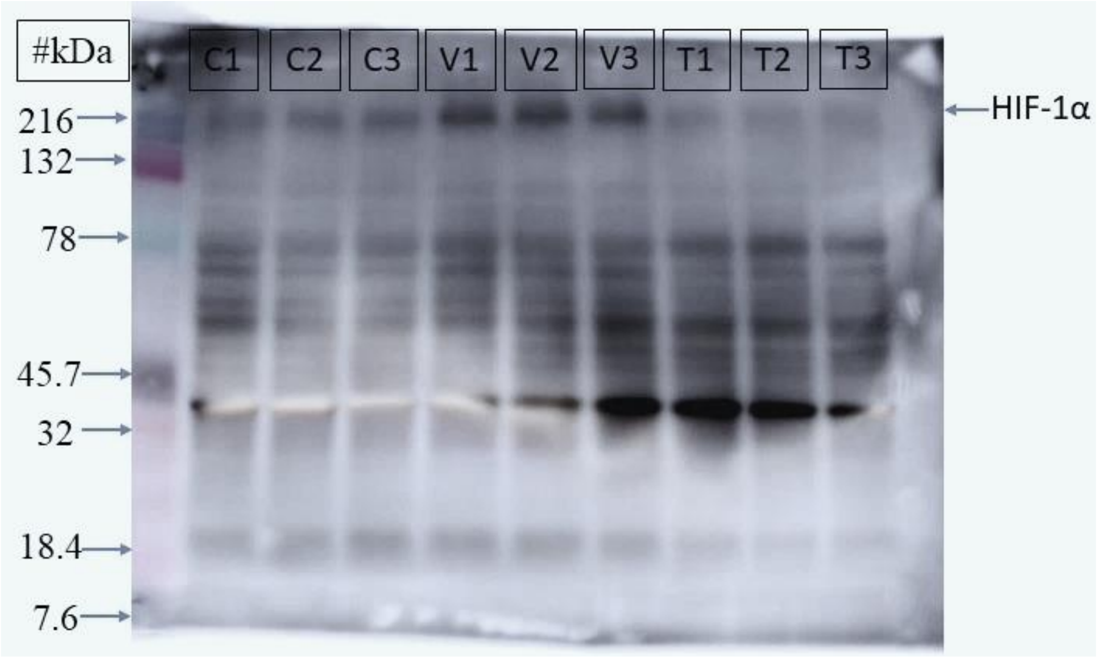

Figure S1. Western blot membrane of HIF-1α protein detected with anti- HIF-1α antibody. Gel-separated proteins were transferred to the PVDF membrane (0.2μm pore size; Millipore, Burlington, MA) by electroblotting (1 hr). Membranes were incubated overnight with a primary antibody (HIF-1α: cat# H1Alpha67, ab1 monoclonal antibody, Abcam, Cambridge, United Kingdom, 1:2000) followed by incubation with a secondary antibody (1:5000 for 2 hr (cat#B0618, Vector Laboratories, CA, United States). The blots were developed with an ECL substrate kit (RPN2106, GE Healthcare, UK). #protein standard marker (molecular weight in kDa, Kaleidoscope, prestained protein standard,7.6 to 216 kDa, BIO-RAD, cat no:161-0324). Blot images were developed, and density was analyzed using Image J software. C1, C2, C3- protein from Control group (sham); V1, V2, V3- Protein from Virus (MHV-1) group; T1, T2, T3- protein from Virus + treatment (MHV-1+SPK) group respectively.

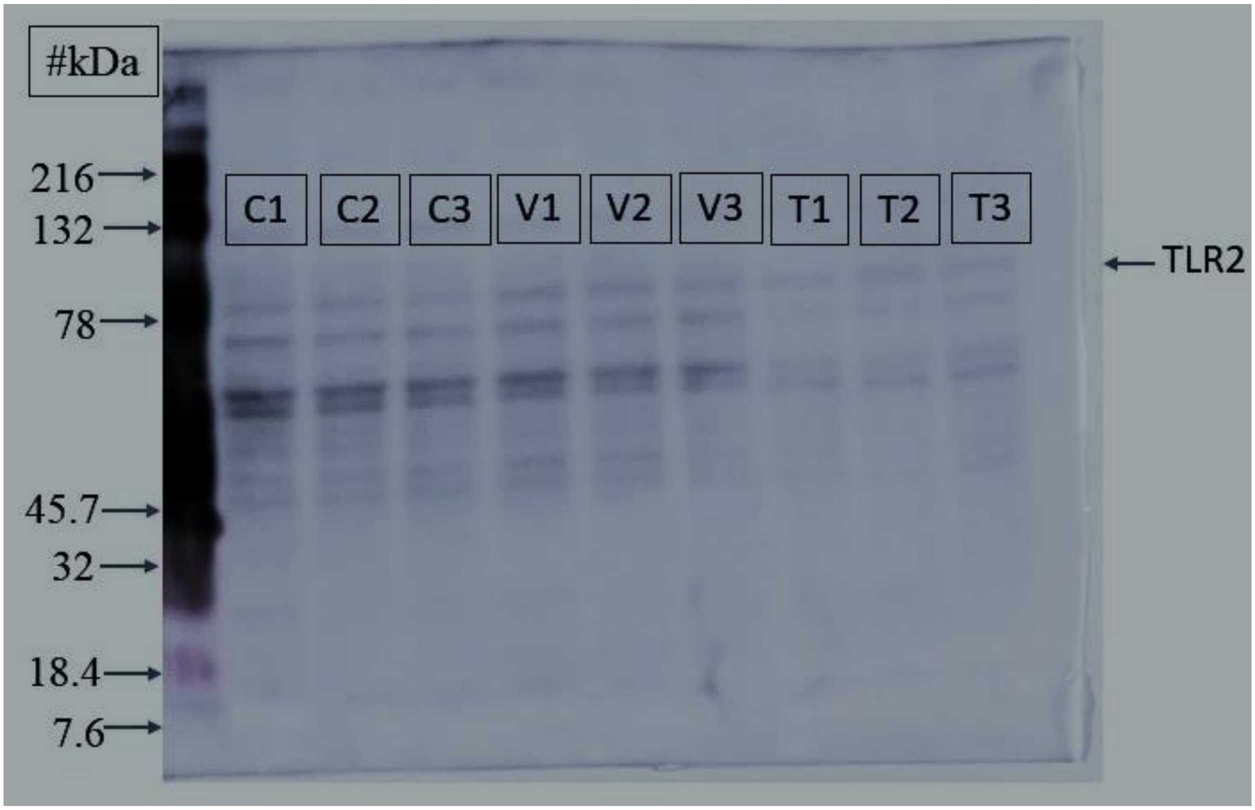

Figure S2. Western blot membrane of TLR2 protein detected with anti-TLR2 antibody. Gel-separated proteins were transferred to the PVDF membrane (0.2µm pore size; Millipore, Burlington, MA) by electroblotting (1 hr). Membranes were incubated overnight with a primary antibody (TLR2: cat# 06-1119 polyclonal antibody, Millipore Sigma, MA, 1:2000) followed by incubation with a secondary antibody (1:5000 for 2 hr (cat#B0618, Vector Laboratories, CA, United States). The blots were developed with an ECL substrate kit (RPN2106, GE Healthcare, UK). #protein standard marker (molecular weight in kDa, Kaleidoscope, prestained protein standard,7.6 to 216 kDa, BIO-RAD, cat no:161-0324). Blot images were developed, and density was analyzed using Image J software. C1, C2, C3- protein from Control group (sham); V1, V2, V3- Protein from Virus (MHV-1) group; T1, T2, T3- protein from Virus + treatment (MHV-1+SPK) group respectively.

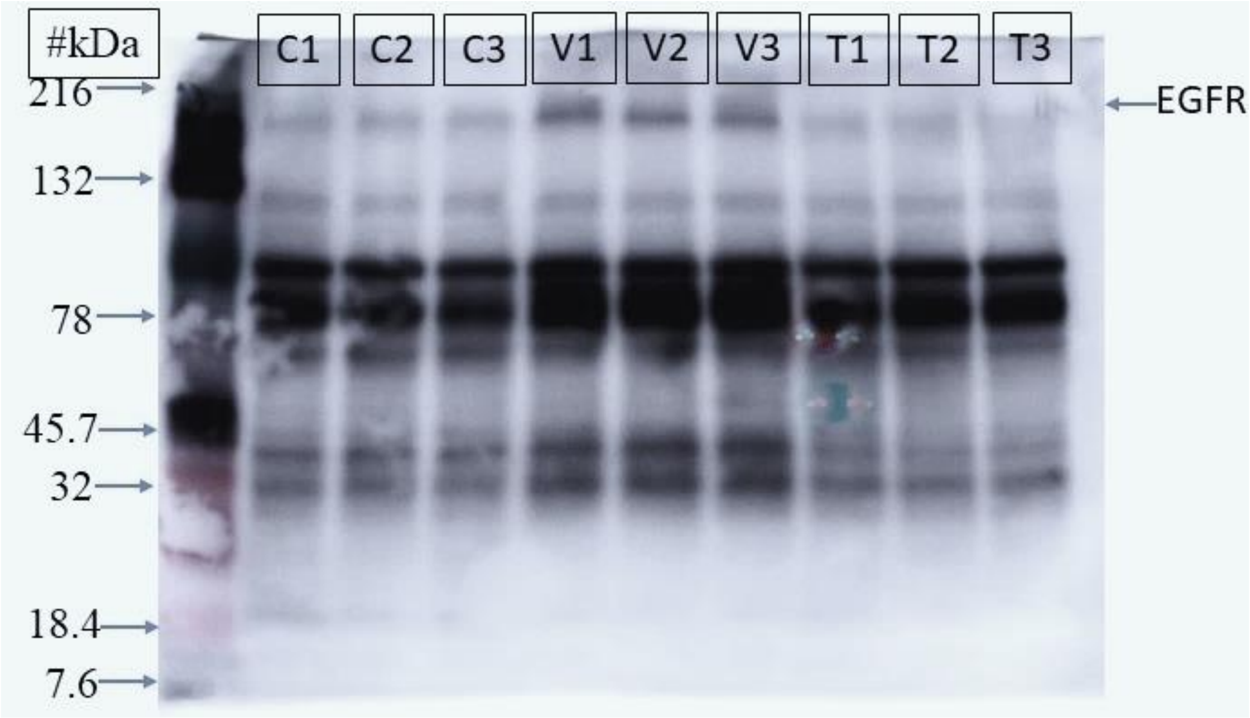

Figure S3. Western blot membrane of EGFR protein detected with anti-EGFR antibody. Gel-separated proteins were transferred to the PVDF membrane (0.2µm pore size; Millipore, Burlington, MA) by electroblotting (1 hr). Membranes were incubated overnight with a primary antibody (EGFR: cat# 05-1047 monoclonal antibody, Millipore Sigma, Burlington, MA, 1:2000) followed by incubation with a secondary antibody (1:5000 for 2 hr (cat#B0618, Vector Laboratories, CA, United States). The blots were developed with an ECL substrate kit (RPN2106, GE Healthcare, UK). #protein standard marker (molecular weight in kDa, Kaleidoscope, prestained protein standard,7.6 to 216 kDa, BIO-RAD, cat no:161-0324). Blot images were developed, and density was analyzed using image J software. C1, C2, C3- protein from Control group (sham); V1, V2, V3- Protein from Virus (MHV-1) group; T1, T2, T3- protein from Virus + treatment (MHV-1+SPK) group respectively.

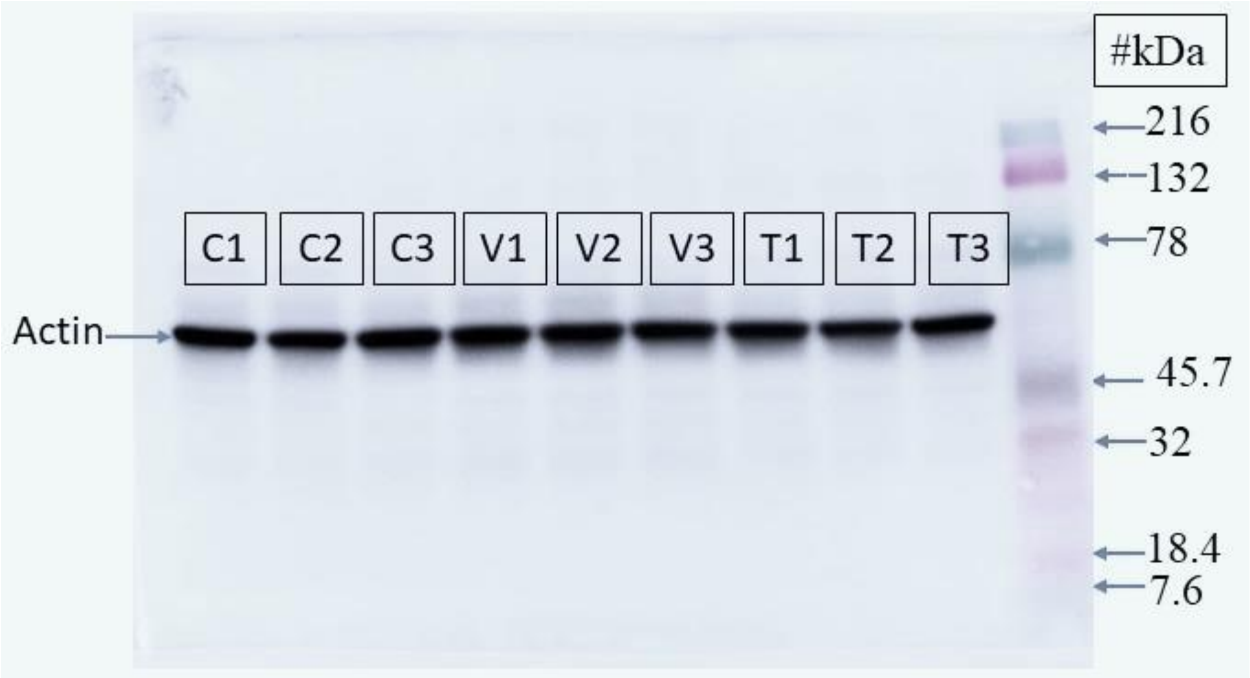

Figure S4. Western blot membrane of β-Actin protein detected with anti-β-Actin antibody. Gel-separated proteins were transferred to the PVDF membrane (0.2μm pore size; Millipore, Burlington, MA) by electroblotting (1 hr). Membranes were incubated overnight with a primary antibody (Actin: cat#B0618, Santacruz Biotechnology, Texas, USA, 1:3000-1:5000) followed by incubation with a secondary antibody (1:5000 for 2 hr (cat#B0618, Vector Laboratories, CA, United States). The blots were developed with an ECL substrate kit (RPN2106, GE Healthcare, UK). #protein standard marker (molecular weight in kDa, Kaleidoscope, prestained protein standard, 7.6 to 216 kDa, BIO-RAD, cat no:161-0324). Blot images were developed, and density was analyzed using Image J software. C1, C2, C3- protein from Control group (sham); V1, V2, V3- Protein from Virus (MHV-1) group; T1, T2, T3- protein from Virus + treatment (MHV-1+SPK) group respectively.

Table S1: Densitometry data of HIF-1α, TLR2 and EGFR from PVDF membrane. The proteins were normalized using β-actin.

Protein	Protein Density		
	Control	MHV-1	MHV-1+SPK
HIF-1α	29169<br>37564<br>37564	68514<br>69802<br>57969	8847<br>9142<br>8945
TLR2	32280<br>25359<br>28541	45015<br>38001<br>33245	16424<br>7454<br>8401
EGFR	12012<br>8640<br>9142	66392<br>48766<br>57018	12012<br>14250<br>9142
β-Actin	118493<br>105503<br>123039	122754<br>124653<br>126478	124651<br>114615<br>129299
